# Development and Preliminary Validation of the Salzburg Emotional Eating Scale

**DOI:** 10.3389/fpsyg.2018.00088

**Published:** 2018-02-06

**Authors:** Adrian Meule, Julia Reichenberger, Jens Blechert

**Affiliations:** ^1^Department of Psychology, University of Salzburg, Salzburg, Austria; ^2^Centre for Cognitive Neuroscience, University of Salzburg, Salzburg, Austria

**Keywords:** emotional eating, positive emotions, negative emotions, arousal, eating behavior, BMI

## Abstract

Existing self-report questionnaires for the assessment of emotional eating do not differentiate between specific types of emotions *and* between increased or decreased food intake in response to these emotions. Therefore, we developed a new measure of emotional eating—the Salzburg Emotional Eating Scale (SEES)—for which higher scores indicate eating more than usual in response to emotions and lower scores indicate eating less than usual in response to emotions. In study 1, a pool of items describing 40 emotional states was used. Factor analysis yielded four factors, which represented both positive (*happiness* subscale) and negative emotions (*sadness, anger*, and *anxiety* subscales). Subsequently, the scale was reduced to 20 items (5 items for each subscale) and its four-factor structure was replicated in studies 2 and 3. In all three studies, internal consistencies of each subscale were α > 0.70 and mean subscale scores significantly differed from each other such that individuals reported the strongest tendency to eat more than usual when being sad and the strongest tendency to eat less than usual when being anxious (sadness > happiness > anger > anxiety). Higher scores on the happiness subscale related to lower scores on the negative emotions subscales, lower body mass index (BMI), and lower eating pathology. In contrast, higher scores on the negative emotions subscales related to lower scores on the happiness subscale, higher BMI, and higher eating pathology. The SEES represents a useful measure for the investigation of emotional eating by increasing both specificity (differentiation between specific emotional states) and breadth (differentiation between increase and decrease of food intake) in the assessment of the emotion–eating relationship.

## Introduction

Emotional eating is often defined as an increase in food intake in order to cope with negative emotions (Macht and Simons, 2011). This type of eating style characterizes several eating-related psychopathologies. For example, negative affect precipitates binge eating episodes in individuals with bulimia nervosa and binge eating disorder (Haedt-Matt and Keel, 2011) and increases desire to eat in obese individuals (van Strien et al., 2016). Although food intake is often used as an emotion regulation strategy, it usually does not reduce negative affect effectively and, thus, the mechanisms that generate and maintain emotional eating are far from being clear (Haedt-Matt et al., 2014). Moreover, it seems that there are numerous moderators that determine the effects of emotions on eating behavior such as the arousal level that accompanies certain emotions or individual differences in eating habits (Macht, 2008).

While the majority of studies on emotional eating have focused on eating to alleviate a negative mood, it has been shown that a positive mood can also result in increased food intake (Cardi et al., 2015). Moreover, while most research has focused on increased food intake, it has been found that experiencing emotions can also result in a decrease in food intake, depending on self-reported emotional eating tendencies (van Strien et al., 2012). A recent review of the literature on self-report measures of emotional eating criticized their lack of predictive validity, pointing to the weak and inconsistent associations with actual food intake in laboratory-based and naturalistic studies (Bongers and Jansen, 2016). One reason for this might be that they only cover a restricted range of relevant aspects of emotional eating. For example, most measures solely include negative emotions and only ask about eating more in response to these emotions, leaving out the possibility of reduced food intake (Bongers and Jansen, 2016).

A range of self-report measures are available for the assessment of emotional eating tendencies such as the Dutch Eating Behavior Questionnaire (DEBQ; van Strien et al., 1986), the Emotional Eating Scale (EES; Arnow et al., 1995), the Emotional Overeating Questionnaire (EOQ; Masheb and Grilo, 2006), the Emotional Appetite Questionnaire (EMAQ; Geliebter and Aversa, 2003), and the Positive-Negative Emotional Eating Scale (PNEES; Sultson et al., 2017). To date, the only self-report questionnaire that does differentiate between positive and negative emotions *and* assesses eating less or eating more in response to these emotions is the EMAQ. However, this scale was not developed via item selection based on empirical data and does not differentiate between different negative emotions (e.g., with different arousal levels; Geliebter and Aversa, 2003). Therefore, we developed a new self-report measure of emotional eating that differentiates between different emotions *and* between decreased or increased food consumption in response to these emotions (Salzburg Emotional Eating Scale, SEES). For this, an item pool of 40 items was created (see method section below and Table 1).

**Table 1:** Factor loadings and item statistics in study 1.

**Item**	**Factor**				***M***	***SD***
	**Happiness**	**Sadness**	**Anger**	**Anxiety**		
1. When I feel lonely, … [Wenn ich mich einsam fühle, …]	–	**0.795**	–	–	3.38	1.03
2. When I am sad, … [Wenn ich traurig bin, …]	–	**0.719**	–	–	2.80	1.19
3. When I am angry, … [Wenn ich verärgert bin, …]	–	–	**0.804**	–	2.90	0.84
4. When I am bored, … [Wenn ich mich langweile, …]	–	**0.692**	–	–	4.07	0.79
5. When I am anxious, … [Wenn ich ängstlich bin, …]	–	–	–	**0.699**	2.44	0.86
6. When I am frustrated, … [Wenn ich frustriert bin, …]	–	**0.669**	–	–	3.44	1.01
7. When I am discouraged, … [Wenn ich mutlos bin, …]	–	0.474	–	–	2.93	0.88
8. When I am upset, … [Wenn ich aufgebracht bin, …]	–	–	**0.651**	–	2.76	0.87
9. When I am worried, … [Wenn ich besorgt bin, …]	–	–	–	**0.532**	2.62	0.96
10. When I am depressed, … [Wenn ich deprimiert bin, …]	–	**0.727**	–	–	3.12	1.21
11. When I am tense, … [Wenn ich angespannt bin, …]	–	–	–	**0.770**	2.51	1.01
12. When I am tired, … [Wenn ich müde bin, …]	–	–	–	–	2.55	0.95
13. When I am irritated, … [Wenn ich gereizt bin, …]	–	–	**0.745**	–	2.91	0.90
14. When I am in despair, … [Wenn ich verzweifelt bin, …]	–	0.489	–	–	2.71	1.09
15. When I am furious, … [Wenn ich wütend bin, …]	–	–	**0.851**	–	2.85	0.95
16. When I am jealous, … [Wenn ich eifersüchtig bin, …]	–	–	**0.498**	–	2.75	0.82
17. When I feel uneasy, … [Wenn ich unsicher bin, …]	–	–	–	**0.410**	2.77	0.82
18. When I feel guilty, … [Wenn ich mich schuldig fühle, …]	–	–	–	–	2.68	0.91
19. When I feel helpless, … [Wenn ich mich hilflos fühle, …]	–	0.430	–	–	2.86	0.92
20. When I am disappointed, … [Wenn ich enttäuscht bin, …]	–	0.534	–	–	3.02	0.96
21. When I am cheerful, … [Wenn ich fröhlich bin, …]	**0.713**	–	–	–	2.98	0.64
22. When I am happy, … [Wenn ich glücklich bin, …]	**0.715**	–	–	–	3.06	0.67
23. When I am satisfied, … [Wenn ich zufrieden bin, …]	0.593	–	–	–	3.06	0.59
24. When I feel confident, … [Wenn ich zuversichtlich bin, …]	**0.687**	–	–	–	2.98	0.52
25. When I am self-assured, … [Wenn ich selbstsicher bin, …]	0.656	–	–	–	2.97	0.58
26. When I am relaxed, … [Wenn ich entspannt bin, …]	0.554	–	–	–	3.08	0.66
27. When I feel playful, … [Wenn ich mich ausgelassen fühle, …]	0.507	–	–	–	3.05	0.71
28. When I am thrilled, … [Wenn ich begeistert bin]	0.655	–	–	–	2.91	0.63
29. When I am enthusiastic, … [Wenn ich enthusiastisch bin, …]	0.666	–	–	–	2.87	0.66
30. When I am in love, … [Wenn ich verliebt bin, …]	0.420	–	–	–	2.48	0.86
31. When I am nervous, … [Wenn ich aufgeregt bin, …]	–	–	–	**0.686**	2.34	0.98
32. When I feel optimistic, … [Wenn ich optimistisch bin, …]	**0.734**	–	–	–	2.97	0.59
33. When I am jolly, … [Wenn ich vergnügt bin, …]	0.625	–	–	–	3.04	0.70
34. When I feel strong, … [Wenn ich mich stark fühle, …]	0.625	–	–	–	2.94	0.73
35. When I am proud, … [Wenn ich stolz bin, …]	**0.703**	–	–	–	3.01	0.66
36. When I am determined, … [Wenn ich entschlossen bin, …]	0.575	–	–	–	2.80	0.62
37. When I feel alert, … [Wenn ich mich wach fühle, …]	0.520	–	–	–	2.93	0.58
38. When I am relieved, … [Wenn ich erleichtert bin, …]	0.461	–	–	–	3.15	0.63
39. When I am unworried, … [Wenn ich unbekümmert bin, …]	0.662	–	–	–	3.04	0.61
40. When I feel secure, … [Wenn ich mich geborgen fühle, …]	0.555	–	–	–	3.15	0.70

*German item wording in square brackets. Five items with the highest factor loadings for each factor (printed in boldface) were selected for the Salzburg Emotional Eating Scale. For reasons of clarity, only factor loadings > 0.400 are displayed. Response categories were: I eat much less than usual (= 1), I eat less than usual (= 2), I eat just as much as usual (= 3), I eat more than usual (= 4), and I eat much more than usual (= 5)*.

In study 1, we explored factor structure of this item pool and items with the highest factor loadings were selected for further refining the scale. We expected that participants would report eating more than usual in response to positive emotions and negative, low arousing emotions (Cardi et al., 2015). In response to negative, high arousing emotions, however, we expected that participants would report eating less than usual (Reichenberger et al., 2018). Based on the findings with the EMAQ (Nolan et al., 2010; Bourdier et al., 2017), we expected that eating more in response to positive emotions would be associated with eating less in response to negative emotions. Furthermore, men were expected to report eating more in response to positive emotions than women, and women were expected to report eating more in response to negative emotions than men (Nolan et al., 2010). Finally, we expected that eating in response to positive emotions would be associated with lower BMI while eating in response to negative emotions would be associated with higher BMI (Nolan et al., 2010; Bourdier et al., 2017).

Studies 2 and 3 aimed at replicating findings about psychometric properties (factor structure, internal consistencies), mean score differences and associations between subscales, and correlates of the scale (sex, BMI). Study 2 also examined associations with other questionnaire measures as a preliminary indication of validity. Specifically, we expected that there would be medium-to-high correlations with similar questionnaire measures on stress- and emotional eating and small correlations with other eating-related questionnaire measures (eating disorder pathology, perceived self-regulatory success in weight regulation). Finally, we expected that there would be no or only small correlations with measures that are not directly related to eating behavior (depressiveness, impulsivity).

## Study 1

### Methods

#### Participants and procedure

The study was approved by the institutional review board of the University of Salzburg. A link to the online survey at www.unipark.com was distributed via e-mail to a mailing list at the University of Salzburg addressing students and staff and via social networks. Participation was voluntary and participants did not receive any compensation. The website was visited 413 times and *n* = 285 participants completed the entire set of questions.

Most participants were women (78.9%, *n* = 225), students (74.7%, *n* = 213), and had German (63.9%, *n* = 182) or Austrian (30.5%, *n* = 87) citizenship. Mean age was *M* = 25.4 years (*SD* = 9.00, Range: 17–67). Mean BMI was *M* = 22.5 kg/m^2^ (*SD* = 4.07, Range: 15.9–43.2). Twenty-eight participants (9.80%) had underweight (BMI < 18.5 kg/m^2^), 208 participants (73.0%) had normal weight (BMI = 18.5–24.9 kg/m^2^), 32 participants (11.2%) had overweight (BMI = 25.0–29.9 kg/m^2^), and 15 participants (5.30%) had obesity (BMI ≥ 30.0 kg/m^2^; BMI data missing for two participants).

#### Measures

A pool of 40 items (20 positive and 20 negative emotions) was generated based on existing questionnaires of emotional eating (DEBQ, EES, EOQ, EMAQ) and the *Positive and Negative Affect Schedule* (Watson et al., 1988). Items are displayed in Table 1. Each item began with the stem *When I am/feel…*, followed by an adjective describing an emotional state. Response categories ranged from *I eat much less than usual* to *I eat much more than usual* (scored from 1 to 5). Items were presented in randomized order in the online survey.

#### Data analyses

Sample size exceeded the minimum 5:1 subjects-to-item ratio necessary for exploratory factor analysis (Costello and Osborne, 2005). Furthermore, the Kaiser-Meyer-Olkin Measure of Sampling Adequacy (KMO = 0.871) and Bartlett's Test of Sphericity [$\chi_{\left(780\right)}^{2}$ = 4390, *p* < 0.001] indicated that data were adequate for conducting an exploratory factor analysis. The number of factors was determined by both parallel analysis and the Minimum Average Partial (MAP) test using the SPSS-syntax provided by O'Connor (2000). Principal Component Analysis was chosen as extraction method and Promax (κ = 4) was selected as rotation method. Internal consistency of factors was evaluated with Cronbach's alpha. Associations between different factor scores and between factor scores and BMI were tested with Pearson correlation coefficients. Sex differences of factor scores were tested with independent samples Mann-Whitney- *U*-tests.

### Results

**Figure 1:**
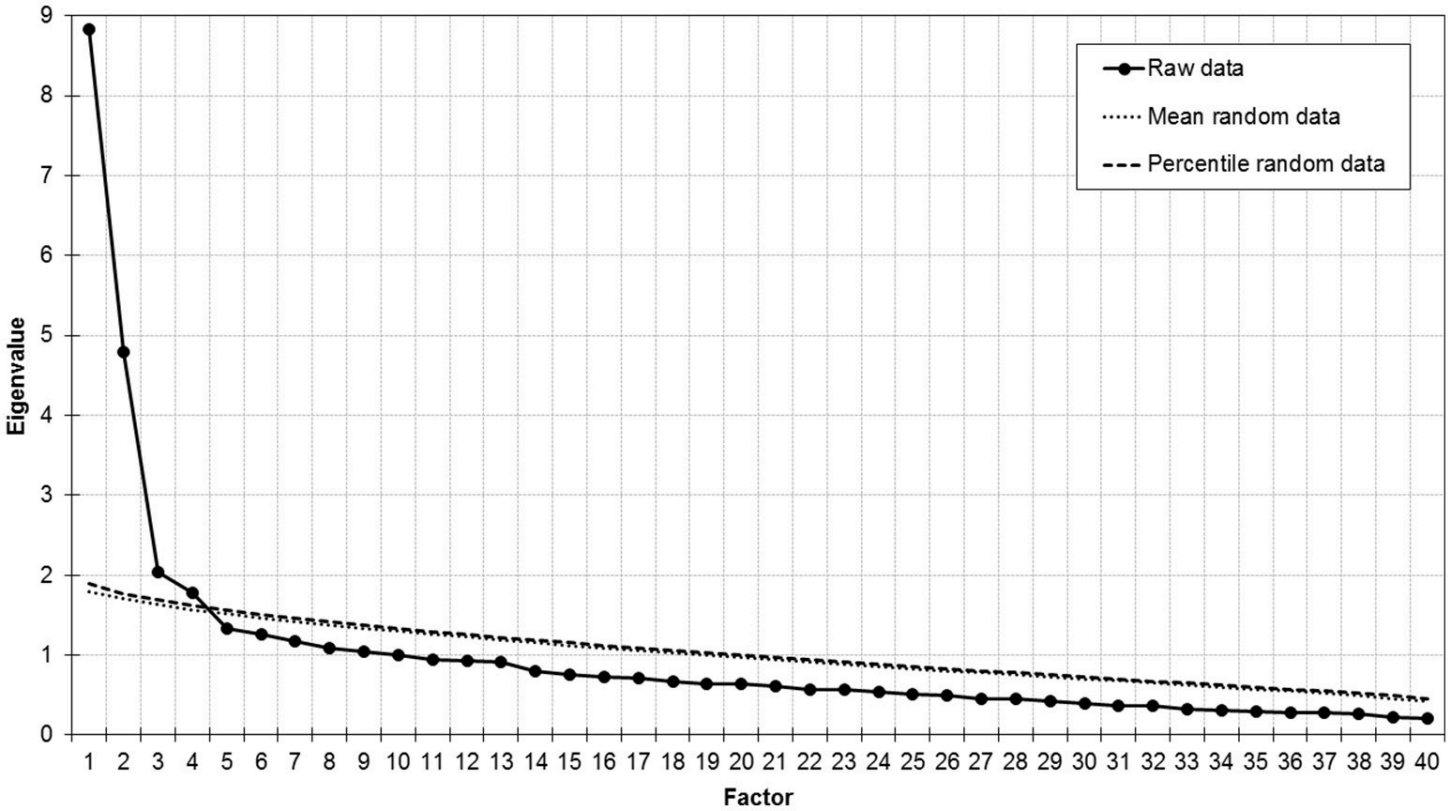
Scree plot and parallel analysis of eigenvalues in study 1.

Parallel analysis (Figure 1) as well as the MAP test (averaged squared partial correlation: component 1 = 0.025, component 2 = 0.010, component 3 = 0.010, component 4 = 0.009, component 5 = 0.010) suggested extraction of four factors, which explained 43.6% of variance. The first factor included items related to positive emotions. The second factor included items related to negative, but low arousing emotions. The third and fourth factor included items related to high arousing emotions such as anger and anxiety (Table 1).

**Figure 2:**
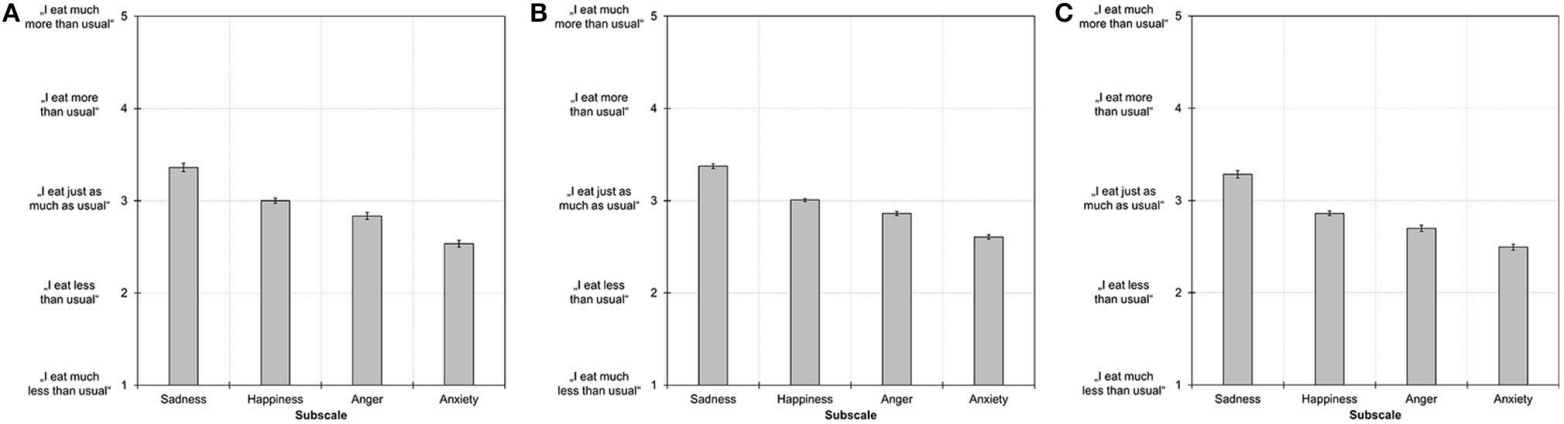
Mean subscale scores on the Salzburg Emotional Eating Scale in study 1 **(A)**, study 2 **(B)**, and study 3 **(C)**. Error bars indicate the standard error of the mean. All subscale scores differed significantly from each other at *p* ≤ 0.001.

In order to reduce the number of items for the SEES, five items with the highest factor loadings were selected for further analyses (Table 1). Thus, this resulted in four subscales that we termed *happiness* (containing the items *cheerful, happy, optimistic, proud, confident*), *sadness* (containing the items *sad, depressed, bored, lonely, frustrated*), *anger* (containing the items *angry, furious, upset, irritated, jealous*), and *anxiety* (containing the items *anxious, worried, tense, uneasy, nervous*). Internal consistencies of these subscales ranged between α = 0.713–0.800 (Table 2). Subscale scores of sadness, anger, and anxiety were positively correlated with each other while scores on happiness were negatively correlated with sadness and anxiety (Table 2). Mean scores on all subscales significantly differed from each other (all *t*s > 3.37, *p*s ≤ 0.001) in the following descending order: sadness > happiness > anger > anxiety (Figure 2A). Men had higher scores (*M* = 3.20, *SD* = 0.54) than women (*M* = 2.95, *SD* = 0.42) on the happiness subscale (*p* < 0.001) and had lower scores (*M* = 3.22, *SD* = 0.66) than women (*M* = 3.40, *SD* = 0.79) on the sadness subscale (*p* = 0.036). Men and women did not differ on anger and anxiety subscale scores (*p*s > 0.080). There were positive correlations between BMI and sadness (*r* = 0.256, *p* < 0.001), anger (*r* = 0.117, *p* = 0.05), and anxiety (*r* = 0.269, *p* < 0.001) subscale scores, but no correlation with the happiness subscale (*r* = 0.011, *p* = 0.856).

**Table 2:** Descriptive statistics and internal consistency of and correlations between the four subscales of the Salzburg Emotional Eating Scale in study 1.

***n* = 285**	***M***	***SD***	**Range**	**1**	**2**	**3**	**4**
1. Happiness	3.00	0.46	1.00–5.00	(α = 0.800)	−0.206^*^	−0.114	−0.193^*^
2. Sadness	3.36	0.77	1.00–5.00	–	(α = 0.773)	0.336^*^	0.344^*^
3. Anger	2.84	0.64	1.00–5.00	–	–	(α = 0.775)	0.384^*^
4. Anxiety	2.54	0.63	1.00–5.00	–	–	–	(α = 0.713)

* *p ≤ 0.001*.

## Study 2

### Methods

#### Participants and procedure

The study was approved by the institutional review board of the University of Salzburg. A link to the online survey at www.unipark.com was distributed via e-mail to student mailing lists at several German and Austrian universities, via social networks, and via a posting on the website of the German version of Psychology Today. Three × 50 € were raffled among participants who completed the survey. The website was visited 1,396 times and *n* = 805 participants completed the entire set of questions. Fifteen participants were excluded from analyses because they answered questions too rapidly (total completion time less than 5 min), leaving a final sample size of *n* = 790.

Most participants were women (82.9%, *n* = 655) and had German (81.3%, *n* = 642) or Austrian (14.2%, *n* = 112) citizenship. The majority of participants were students (79.6%, *n* = 629), employed (11.4%, *n* = 90), or pupils (4.70%, *n* = 37). Mean age was *M* = 24.7 years (*SD* = 6.79, Range: 15–65). Mean BMI was *M* = 22.3 kg/m^2^ (*SD* = 3.93, Range: 15.0–50.9). Seventy-six participants (9.60%) had underweight (BMI < 18.5 kg/m^2^), 583 participants (73.9%) had normal weight (BMI = 18.5–24.9 kg/m^2^), 92 participants (11.7%) had overweight (BMI = 25.0–29.9 kg/m^2^), and 38 participants (4.80%) had obesity (BMI ≥ 30.0 kg/m^2^; BMI data missing for one participant).

#### Measures

##### Salzburg emotional eating scale (SEES)

The 20-item SEES derived in study 1 with five items per subscale was used. Items were presented in randomized order in the online survey. Internal consistencies of SEES subscales ranged between α = 0.732–0.819 (Table 3).

**Table 3:** Descriptive statistics and internal consistency of and correlations between the four subscales of the Salzburg Emotional Eating Scale in study 2.

***n* = 790**	***M***	***SD***	**Range**	**1**	**2**	**3**	**4**
1. Happiness	3.01	0.47	1.20–5.00	(α = 0.819)	−0.405^*^	−0.285^*^	−0.355^*^
2. Sadness	3.37	0.74	1.20–5.00	–	(α = 0.768)	0.473^*^	0.520^*^
3. Anger	2.86	0.59	1.00–5.00	–	–	(α = 0.754)	0.487^*^
4. Anxiety	2.61	0.65	1.00–5.00	–	–	–	(α = 0.732)

* *p < 0.001*.

##### Salzburg stress eating scale (SSES)

The SSES (Meule et al., 2018a) is a ten-item questionnaire for measuring eating in response to stress. Response categories range from *I eat much less than usual* to *I eat much more than usual* (scored from 1 to 5). Thus, higher values represent eating more when stressed while lower values indicate eating less when stressed. Internal consistency was α = 0.899 in the current study.

##### Dutch eating behavior questionnaire (DEBQ)—emotional eating subscale

The emotional eating subscale of the DEBQ (van Strien et al., 1986; Grunert, 1989) is a ten-item questionnaire for measuring eating in response to negative emotions. Response categories range from *never* to *very often* (scored from 1 to 5). Thus, higher values indicate the frequency of eating more when experiencing negative emotions. Internal consistency was α = 0.909 in the current study.

##### Perceived self-regulatory success in dieting scale (PSRS)

The PSRS (Meule et al., 2012) is a three-item questionnaire for measuring how successful individuals are in watching their weight, in losing weight, and how difficult it is for them to stay in shape. Response categories are anchored *not successful/not difficult* and *very successful/very difficult* (scored from 1 to 7). Thus, higher values indicate higher perceived self-regulatory success in weight regulation. Internal consistency was α = 0.696 in the current study.

##### Eating disorder examination-questionnaire 8 (EDE-Q8)

The EDE-Q8 (Kliem et al., 2016) is an eight-item short form of the EDE-Q for measuring eating disorder psychopathology in the past 28 days. Response categories range from *no days/never/not at all* to *every day/everytime/markedly* (scored from 0 to 6). Thus, higher values indicate higher eating disorder psychopathology. Internal consistency was α = 0.913 in the current study.

##### Center for epidemiologic studies depression scale—short form (CES-D)

The short form of the CES-D (Radloff, 1977; Hautzinger et al., 2012) is a 15-item questionnaire for measuring depressive symptoms in the past seven days. Response categories range from *rarely or none of the time* to *most or all of the time* (scored from 0 to 3). Thus, higher values indicate higher depressiveness. Internal consistency was α = 0.911 in the current study.

##### Barratt impulsiveness scale—short form (BIS-15)

The BIS-15 (Spinella, 2007; Meule et al., 2011) is a 15-item short form of the BIS-11 for measuring trait impulsivity. Response categories range from *rarely/never* to *almost always/always* (scored from 1 to 4). Thus, higher values indicate higher impulsivity. Internal consistencies were α = 0.670 (attentional impulsivity subscale), α = 0.734 (motor impulsivity subscale), and α = 0.794 (non-planning impulsivity subscale) in the current study.

#### Data analyses

A confirmatory factor analysis was computed with Amos 24 (IBM SPSS, Chicago) to test the four-factor structure of the SEES found in study 1. Maximum likelihood estimation was used, fixing the factor loading of the first items of every subscale to 1. According to the recommendations of Hu and Bentler (1999), model fit was evaluated by two fit indices: the comparative fit index (CFI), with 0.90 ≤ CFI < 0.95 indicating acceptable fit and CFI ≥ 0.95 indicating good fit, and the root mean square error of approximation (RMSEA), with 0.05 < RMSEA ≤ 0.08 indicating acceptable fit and RMSEA ≤ 0.05 indicating good fit. In order to evaluate whether factor structure of the SEES varied between female and male participants, we tested measurement invariance at three levels: configural, factor loading and intercept invariance. Measurement invariance across sex was evaluated according to recommendations by Chen (2007). Specifically, a χ^2^ difference test can be used for statistical comparison between nested models, but is almost always large and statistically significant with complex models and large samples and, thus, an impractical and unrealistic criterion for measurement invariance (Chen et al., 2005). Therefore, model fit changes were examined and decreases in CFI ≤ 0.010 or increases in RMSEA of ≤ 0.015 were considered to indicate measurement invariance (Chen, 2007). Associations between SEES subscale scores, BMI, and scores on the other questionnaires were examined with correlational analyses. Sex differences of SEES subscale scores were tested with independent samples Mann-Whitney- *U*-tests.

#### Results

**Figure 3:**
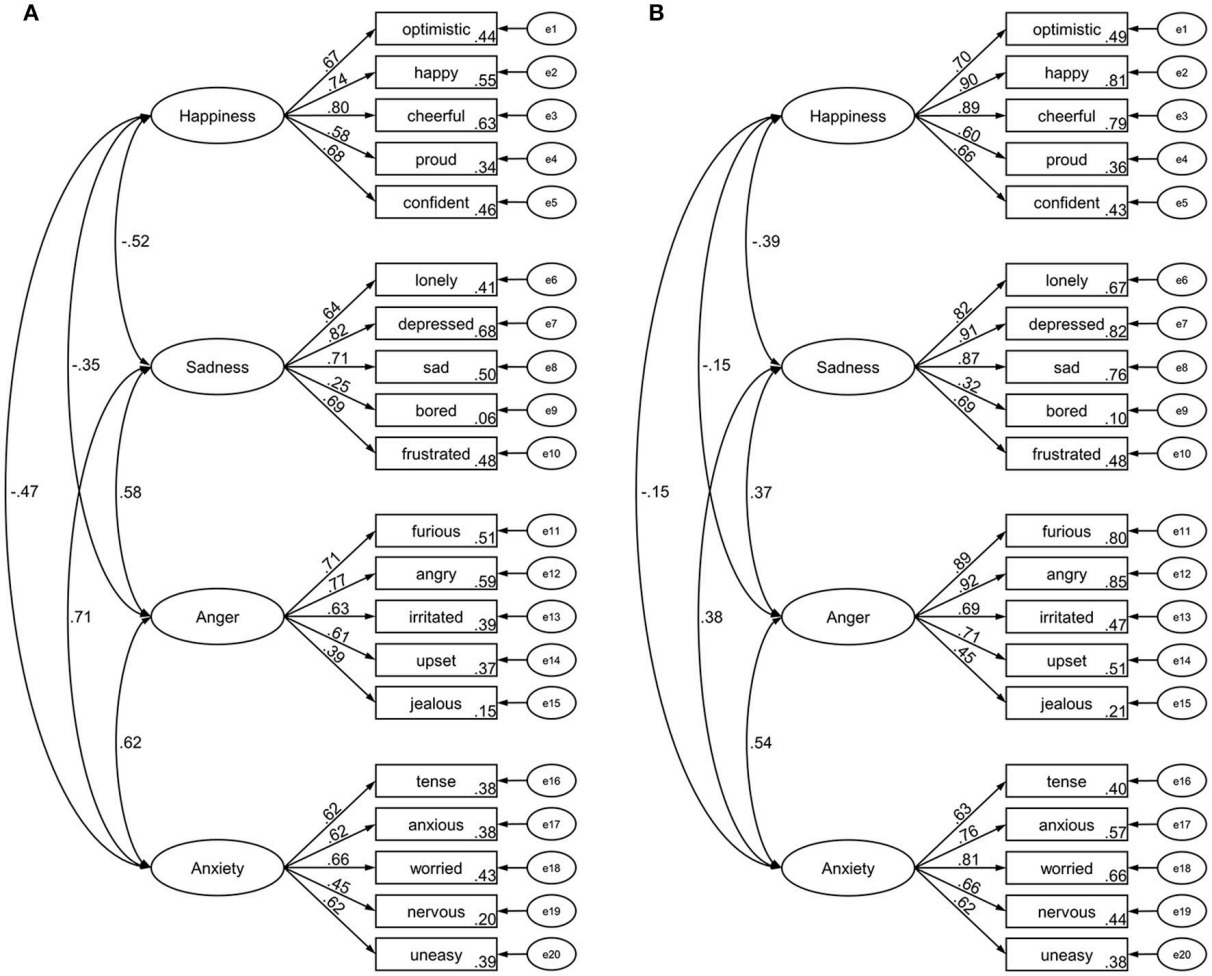
Standardized factor loadings and latent factor intercorrelations of the Salzburg Emotional Eating Scale in study 2 **(A)** and study 3 **(B)**.

The four-factor structure had acceptable model fit (CFI = 0.932, RMSEA = 0.051) and standardized estimates are depicted in Figure 3A. Model fit changes between the configural invariance model (CFI = 0.917, RMSEA = 0.040) and the factor loading invariance model (CFI = 0.917, RMSEA = 0.039) indicated sex invariance for the factor score estimates (ΔCFI = 0.000, ΔRMSEA = 0.001). Similarly, model fit changes between the intercept invariance model (CFI = 0.906, RMSEA = 0.040) and the factor loading model (ΔCFI = 0.011, ΔRMSEA = 0.001) indicated sex invariance for the intercepts.

Subscale scores of sadness, anger, and anxiety were positively correlated with each other while scores on happiness were negatively correlated with sadness, anger, and anxiety scores (Table 3). Similar to study 1, mean scores on all subscales significantly differed from each other (all *t*s > 4.81, *p*s < 0.001) in the following descending order: sadness > happiness > anger > anxiety (Figure 2B). Similar to study 1, men had higher scores (*M* = 3.14, *SD* = 0.44) than women (*M* = 2.98, *SD* = 0.47) on the happiness subscale (*p* < 0.001) and had lower scores (*M* = 3.16, *SD* = 0.65) than women (*M* = 3.42, *SD* = 0.75) on the sadness subscale (*p* < 0.001). In addition, men had lower scores (*M* = 2.75, *SD* = 0.50) than women (*M* = 2.88, *SD* = 0.61) on the anger subscale (*p* = 0.023). Men and women did not differ on anxiety subscale scores (*p* = 0.709).

Scores on the happiness subscale were negatively correlated with BMI, stress eating, emotional eating, eating disorder pathology, depressiveness, and non-planning impulsivity, and positively correlated with perceived self-regulatory success in weight regulation (Table 4). In contrast, scores on the sadness, anger, and anxiety subscales were positively correlated with BMI, stress eating, emotional eating, and eating disorder pathology, and negatively correlated with perceived self-regulatory success in weight regulation (Table 4). In addition, scores on the sadness subscale were positively correlated with depressiveness and attentional impulsivity (Table 4).

**Table 4:** Correlations of scores on the Salzburg Emotional Eating Scale and body mass index as well as questionnaire measures in study 2.

	**Salzburg Emotional Eating Scale subscale**			
	**Happiness**	**Sadness**	**Anger**	**Anxiety**
Body mass index (kg/m^2^)	−0.203^***^	0.205^***^	0.085^*^	0.201^***^
Salzburg Stress Eating Scale	−0.390^***^	0.658^***^	0.519^***^	0.696^***^
Dutch Eating Behavior Questionnaire—emotional eating	−0.316^***^	0.654^***^	0.415^***^	0.403^***^
Perceived Self-Regulatory Success in Dieting Scale	0.155^***^	−0.287^***^	−0.127^***^	−0.186^***^
Eating Disorder Examination-Questionnaire 8	−0.222^***^	0.206^***^	0.131^***^	0.157^***^
Center for Epidemiologic Studies Depression Scale—short form	−0.108^**^	0.120^**^	0.050	0.025
Barratt Impulsiveness Scale				
Attentional impulsivity	−0.035	0.122^**^	0.019	0.037
Motor impulsivity	−0.058	0.060	0.014	0.048
Non-planning impulsivity	−0.078^*^	0.046	−0.001	0.048

* *p < 0.050*,

** *p < 0.010*,

*** *p < 0.001*.

## Study 3

### Methods

#### Participants and procedure

The study was approved by the institutional review board of the University of Salzburg. A link to the online survey at www.limesurvey.org was distributed via e-mail to student mailing lists at the University of Salzburg. To broaden the age range as compared to studies 1 and 2, these included students from the university's 55-PLUS program, which is an educational opportunity for older adults at the university. Furthermore, adolescent participants were recruited at a local high school. Participation was voluntary and participants did not receive any compensation. The website was visited 623 times and *n* = 450 participants completed the study.

Most participants were women (74.4%, *n* = 335) and had Austrian (50.9%, *n* = 229) or German (42.7%, *n* = 192) citizenship. The majority of participants indicated their occupation as student (29.1%, *n* = 131), employed (26.4%, *n* = 119), or other (32.2%, *n* = 145). Mean age was *M* = 33.5 years (*SD* = 18.2, Range: 14–86). Mean BMI was *M* = 23.8 kg/m^2^ (*SD* = 4.91, Range: 15.6–50.2). Thirty-four participants (7.60%) had underweight (BMI < 18.5 kg/m^2^), 291 participants (64.7%) had normal weight (BMI = 18.5–24.9 kg/m^2^), 79 participants (17.6%) had overweight (BMI = 25.0–29.9 kg/m^2^), and 46 participants (10.2%) had obesity (BMI ≥ 30.0 kg/m^2^).

#### Measures

The 20-item SEES was used and internal consistencies ranged between α = 0.820–0.871 (Table 5).

**Table 5:** Descriptive statistics and internal consistency of and correlations between the four subscales of the Salzburg Emotional Eating Scale in study 3.

***n* = 450**	***M***	***SD***	**Range**	**1**	**2**	**3**	**4**
1. Happiness	2.86	0.55	1.00–5.00	(α = 0.871)	−0.315^*^	−0.111^*^	−0.112^*^
2. Sadness	3.29	0.83	1.20–5.00	–	(α = 0.855)	0.353^*^	0.323^*^
3. Anger	2.70	0.70	1.00–5.00	–	–	(α = 0.856)	0.565^*^
4. Anxiety	2.49	0.69	1.00–5.00	–	–	–	(α = 0.820)

* *p < 0.050*.

#### Data analyses

Data analyses regarding factor structure and measurement invariance of the SEES, differences and associations between subscales, and relationships with sex and BMI were identical to study 2.

### Results

The four-factor structure had acceptable model fit (CFI = 0.917, RMSEA = 0.073) and standardized estimates are depicted in Figure 3B. Model fit changes between the configural invariance model (CFI = 0.898, RMSEA = 0.058) and the factor loading invariance model (CFI = 0.895, RMSEA = 0.058) indicated sex invariance for the factor score estimates (ΔCFI = 0.003, ΔRMSEA = 0.000). Similarly, model fit changes between the intercept invariance model (CFI = 0.877, RMSEA = 0.061) and the factor loading model (ΔCFI = 0.018, ΔRMSEA = 0.003) indicated sex invariance for the intercepts.

Similar to studies 1 and 2, subscale scores of sadness, anger, and anxiety were positively correlated with each other while scores on happiness were negatively correlated with sadness, anger, and anxiety scores (Table 5). Again, mean scores on all subscales significantly differed from each other (all *t*s > 3.73, *p*s < 0.001) in the following descending order: sadness > happiness > anger > anxiety (Figure 2C). Similar to studies 1 and 2, men had higher scores (*M* = 3.04, *SD* = 0.54) than women (*M* = 2.80, *SD* = 0.53) on the happiness subscale (*p* < 0.001) and lower scores (*M* = 3.13, *SD* = 0.70) than women (*M* = 3.34, *SD* = 0.87) on the sadness subscale (*p* = 0.011). In addition, men had higher scores (*M* = 2.65, *SD* = 0.60) than women (*M* = 2.44, *SD* = 0.71) on the anxiety subscale (*p* = 0.003). Men and women did not differ on anger subscale scores (*p* = 0.784). BMI correlated positively with sadness (*r* = 0.147, *p* = 0.002) and anxiety (*r* = 0.141, *p* = 0.003) subscale scores, negatively with happiness subscale scores (*r* = −0.165, *p* < 0.001), and did not correlate with anger subscale scores (*r* = 0.041, *p* = 0.381).

## Discussion

Progress in the research on the conceptual foundations of emotional eating, its potential mechanisms and its clinical correlates requires continuous refinement of the respective psychometric scales. The current studies document the development and preliminary validation of a new self-report measure of emotional eating—the SEES—which extends previous scales by detailing specific emotions and differentiating emotional over- and undereating. Item reduction from a pool of 40 emotional states resulted in a 20-item scale with four subscales, which were invariant across sex and had acceptable-to-good internal consistencies. Scores on these subscales represent self-reported eating in response to positive emotions (*happiness*), negative but low arousal emotions (*sadness*), and negative but high arousing emotions (*anger* and *anxiety*). These four affective states are consistent with the four most often measured emotions in emotion research (Weidman et al., 2017). Higher scores represent increased food intake, medium scores represent unchanged food intake, and lower scores represent decreased food intake.

Subscale scores significantly differed from each other: there was an overall tendency to report eating more than usual when experiencing sadness, eating just as much as usual when experiencing happiness, and eating less than usual when experiencing anger or anxiety (Figure 2). These differences might be attributable to different levels of bodily arousal that accompanies these emotions (Macht, 2008) and respective neuroendocrine changes (Torres and Nowson, 2007). Therefore, results point to specific mappings of emotion type on intake type (e.g., sadness to overeating, anger/anxiety to undereating) that—if collapsed by a measure—might mask or fully occlude any relationship and, thus, lead to inconsistent or contradictory findings.

In line with findings with the EMAQ, men reported to eat more when being happy whereas women reported to eat more when being sad. Moreover, eating in response to positive emotions was negatively correlated with eating in response to negative emotions and, similarly, correlates of these subscales diverged (Nolan et al., 2010; Bourdier et al., 2017). Although results of all three studies were not entirely consistent, an overall picture emerged such that eating more in response to happiness was associated with having a lower BMI, reporting lower eating pathology, and higher perceived success in weight regulation. Thus, it appears that such “happy overeating” represents a functional, healthy eating style that may reflect an intuitive change in eating behavior associated with appropriate perception of bodily signals (Herbert et al., 2013). Moreover, higher scores on the happiness subscale were associated with eating less in response to negative emotions, which may indicate that “happy overeating” and “unhappy undereating” might be two sides of the same coin and together associated with positive eating- and weight-related outcomes. This interpretation, however, needs to consider a person's body weight. For example, we have preliminary data available showing that inpatients with anorexia nervosa have significantly higher scores on the happiness subscale and lower scores on the sadness subscale than normal-weight control participants (Meule et al., 2018b). This suggests that the “happy overeating”–“unhappy undereating” combination can also reflect higher eating disorder severity in some individuals, particularly in those with underweight.

In contrast, eating more in response to negative emotions was associated with having a higher BMI, reporting higher eating pathology, and lower perceived success in weight regulation. Again, the flipside of this pattern was eating less in response to happiness, suggesting that the configuration of “unhappy overeating” and “happy undereating” is a more dysfunctional, unhealthy eating pattern that relates to unfavorable eating- and weight-related outcomes. Thus, these findings replicate and extend findings about correlates of eating in response to positive and negative emotions (Geliebter and Aversa, 2003; Nolan et al., 2010; Bourdier et al., 2017), providing preliminary support for validity and usefulness of the SEES.

Although the SEES allows for a more fine-grained analysis of emotional effects on eating, it still relies on self-report, which can potentially be biased. Specifically, the questionnaire requires that participants have significant insight of their day-to-day fluctuations in affect and its influence on food intake. For example, it has been suggested that individuals scoring high on self-report measures of emotional eating may overestimate how much they actually eat in response to certain emotions or may simply attribute their overeating to negative affect retrospectively (Royal and Kurtz, 2010; Adriaanse et al., 2016). Thus, validity of the SEES may be investigated in future studies that examine relationships with implicit measures (e.g., an emotional eating-related implicit association task; Bongers et al., 2013), with food intake after emotion induction in the laboratory, or with emotional eating assessed in daily life (e.g., with a combination of ecological momentary assessment and dietary assessment such as the Automated Self-Administered 24-h dietary assessment tool; Subar et al., 2012). Furthermore, our data are based on samples, which entail common limitations of online-based research (e.g., self-selection, completing the questionnaire multiple times). Thus, it is necessary to examine psychometric properties (e.g., measurement invariance across different age groups) and correlates of the SEES in more representative samples, which also include, for example, a higher proportion of individuals with lower education.

In accordance with other reports (Bongers and Jansen, 2016), our data suggest that focusing solely on increased food intake in response to negative emotions only covers a small proportion of what can be termed emotional eating. Specifically, different emotions have different effects on food intake and these have dissociable correlates. For example, “happy overeating” (or “unhappy undereating”) as opposed to “happy undereating” (or “unhappy overeating”) appears to be an adaptive, functional behavior in most individuals. Thus, we suggest that traditional definitions of the term *emotional eating* need to be opened up to all possible combinations of emotion and intake types such that emotional eating can be defined as *any alteration in food intake* (which can include eating less or eating more than usual) *in response to affective states* (which can include positive and negative emotions). By providing a fine-grained assessment of emotional eating that takes these aspects into account, the SEES opens exciting new avenues for research on the mechanisms and clinical correlates of emotional eating, not only in the psychometric domain but also in experimental and naturalistic studies.

## Author contributions

AM and JB: Conceived the studies; AM and JR: Conducted the statistical analyses; AM: Drafted the manuscript; JR and JB revised the manuscript.

### Conflict of interest statement

The authors declare that the research was conducted in the absence of any commercial or financial relationships that could be construed as a potential conflict of interest.
